# TIGIT, the Next Step Towards Successful Combination Immune Checkpoint Therapy in Cancer

**DOI:** 10.3389/fimmu.2021.699895

**Published:** 2021-07-22

**Authors:** Zhouhong Ge, Maikel P. Peppelenbosch, Dave Sprengers, Jaap Kwekkeboom

**Affiliations:** Department of Gastroenterology and Hepatology, Erasmus University Medical Center (MC), Rotterdam, Netherlands

**Keywords:** immunotherapy, T cells, NK cells, CD226, CD155

## Abstract

T cell immunoreceptor with Ig and ITIM domains (TIGIT) is an inhibitory receptor expressed on several types of lymphocytes. Efficacy of antibody blockade of TIGIT in cancer immunotherapy is currently widely being investigated in both pre-clinical and clinical studies. In multiple cancers TIGIT is expressed on tumor-infiltrating cytotoxic T cells, helper T cells, regulatory T cells and NK cells, and its main ligand CD155 is expressed on tumor-infiltrating myeloid cells and upregulated on cancer cells, which contributes to local suppression of immune-surveillance. While single TIGIT blockade has limited anti-tumor efficacy, pre-clinical studies indicate that co-blockade of TIGIT and PD-1/PD-L1 pathway leads to tumor rejection, notably even in anti-PD-1 resistant tumor models. Among inhibitory immune checkpoint molecules, a unique property of TIGIT blockade is that it enhances not only anti-tumor effector T-cell responses, but also NK-cell responses, and reduces the suppressive capacity of regulatory T cells. Numerous clinical trials on TIGIT-blockade in cancer have recently been initiated, predominantly combination treatments. The first interim results show promise for combined TIGIT and PD-L1 co-blockade in solid cancer patients. In this review, we summarize the current knowledge and identify the gaps in our current understanding of TIGIT’s roles in cancer immunity, and provide, based on these insights, recommendations for its positioning in cancer immunotherapy.

## Highlights

- TIGIT blockade synergizes with PD-1/PD-L1 blockade to enhance anti-tumor effects of CD8^+^ T cells.
- TIGIT blockade does not only reinvigorate anti-tumor T cell responses but also enhances anti-tumor NK cell responses.
- TIGIT blockade reduces the suppressive capacity of tumor-infiltrating regulatory T cells.

## Introduction

Therapeutic immune checkpoint inhibitors (ICIs) aimed to restore the functionality of tumor-specific T-cells to combat malignant tumors have been shown to be effective in many cancer types. Specifically, anti-PD-1/PD-L1 therapies have been approved by FDA for treating more than ten cancer entities, including melanoma, non-small cell lung carcinoma (NSCLC), advanced hepatocellular carcinoma (HCC) and all types of deficient mismatch repair(dMMR) tumors (1–3).

However, only a subset of patients with these types of cancer showed durable clinical responses (4, 5), and numerous other types of cancer, among which MMR-proficient CRC, are resistant to anti-PD-1/PD-L1 therapies (6, 7), suggesting that in these patients mechanisms are present that limit the effects of anti-PD-1/PD-L1 antibodies. An immunosuppressive tumor microenvironment, a paucity of T cells in tumors (so called immunologically “cold” tumors) and low tumor-mutational burden have all been suggested to contribute to the observed suboptimal therapeutic effects of anti-PD-1/PD-L1 therapies (8). In addition, although the PD-1/PD-L1 axis plays a central role in regulating T cell functions, there are many other co-inhibitory receptor-ligand interactions that can restrain anti-tumor functions of CD8^+^ T cells in the tumor micro-environment, either directly or indirectly. These co-inhibitory receptors include T cell immunoglobulin mucin domain 3 (TIM3) (9), lymphocyte-activation gene 3 (LAG3) (10), cytotoxic T-lymphocyte associated protein 4 (CTLA-4) (11) and T cell immunoreceptor with Ig and ITIM domains (TIGIT) (12). Their ligands are expressed on cancer cells and/or immune cells in many tumors. These inhibitory receptor-ligand interactions collaborate to achieve fine tuning of T-cell functions throughout the different phases of T-cell activation, and can contribute to resistance of tumors against PD-1/PD-L1 blockade.

In preclinical and subsequent clinical studies, it has been demonstrated that co-blockade of PD-1 and a second co-inhibitory receptor is able to augment the antitumor immunity versus single PD-1 blockade. Indeed, blockade of both PD-1 and CTLA-4 showed improved clinical efficacy in patients with, amongst others, melanoma (13) and advanced non-small-cell lung cancer (14) and hepatocellular carcinoma (15). However, combine PD-1 and CTLA-4 blockade is hampered by a high frequency of severe immune-related systemic adverse effects (16), therefore other less toxic combinations are urgently needed. Theoretically, TIGIT-blockade may be an interesting alternative for CTLA-4 blockade, because TIGIT knockout mice do not develop autoimmunity (17, 18), while CTLA-4 knockout mice die within 2-3 weeks due to severe autoimmunity (19).

Here we review the current knowledge of the immunomodulatory activity of TIGIT in anti-cancer immunity and its potential as a target for cancer immune checkpoint therapy. We identify gaps in our current understanding of TIGIT’s roles in cancer immunity that require further study and, based on current insights, we provide recommendations for its positioning in cancer immune checkpoint therapy.

## TIGIT Structure, Expression, and Ligands

TIGIT [also identified as WUCAM (20), VSTM3 (17)] is a co-inhibitory molecule that was first identified in 2009 (21). Similar to LAG3 and TIM3, TIGIT belongs to the immunoglobulin superfamily (22). It consists of an extracellular immunoglobulin variable (IgV) domain, a type 1 transmembrane domain and a cytoplasmic tail with an immunoreceptor tyrosine-based inhibitory motif (ITIM) and an Ig tail-tyrosine (ITT)-like motif (21–24). TIGIT is exclusively expressed on lymphocytes, including CD8^+^ T cells, memory and regulatory CD4^+^ T cells, follicular CD4^+^ T cells and NK cells (20–22).

TIGIT binds to at least two nectin family members, CD155 (PVR, Necl-5) and CD112 (PVRL2, Nectin-2), but with much higher affinity to CD155 (Kd=1-3 nM) than to CD112 (Kd unmeasurable) (21). TIGIT shares these ligands with two other receptors; CD226 (DNAM-1) and CD96 (TACTILE), which deliver co-stimulatory and co-inhibitory signals, respectively. TIGIT competes with the costimulatory receptor CD226 for binding to CD155, but TIGIT has a higher affinity for CD155 (Kd=1-3 nM) compared to CD226 (Kd=119 nM) (21). A recent paper shows that TIGIT also binds to nectin-4 and that nectin-4 interacts only with TIGIT, and not with CD226 and CD96 (25), but little is known about the functional role of TIGIT-nectin-4 interaction in anti-tumor immunity.

CD155 is expressed on monocytes, dendritic cells (DCs), fibroblasts and endothelial cells, both in healthy tissues and in tumors. Additionally, CD155 is over-expressed on cancer cells in human malignancies including colon cancer (26), lung adenocarcinoma (27), melanoma (28), pancreatic cancer (29), glioblastoma (30) and hepatocellular carcinoma (31). Apart from its normal function in establishing cell-cell adhesion (32, 33), it also functions as the receptor of polio virus (34). Expression of CD155 on tumor cells facilitates tumor cell growth and migration by tumor-intrinsic mechanisms (30) and its overexpression is correlated with tumor progression and unfavorable prognosis (27–29). However, loss of CD155 on tumor cells not only reduces tumor growth by tumor-intrinsic mechanisms, but also improves response to anti-PD-1 antibody therapy in mouse tumor models (35). In addition, high CD155 expression in tumors is associated with resistance to anti-PD-1 therapy in metastatic melanoma patients (36). Moreover, PVRL1 (CD111, nectin-1) promotes TIGIT-mediated T cell suppression by stabilizing CD155 on the surface of HCC tumor cells, and PVRL1 knockdown in tumor cells in an HCC mouse model resulted in reduced tumor growth in a CD8^+^ T cell-dependent manner (37). High expression of CD155 on tumor cells promotes dysfunctionality of tumor infiltrating CD8^+^ T cells by driving internalization and proteasomal degradation of the co-stimulatory receptor CD226 in CD8^+^ T cells in mouse and human tumors (38). These data show that CD155 expression in tumors has dual tumor-promoting effects, both tumor-intrinsic and by inhibition of anti-tumor immunity.

CD112 is expressed by DCs and monocytes (39) and over-expressed in different types of cancers such as acute myeloid leukemia (40), multiple myeloma (41–43) and epithelial cancers (44, 45). CD112 has higher affinity for CD112R (PVRIG) than for TIGIT, and suppresses T cells mainly *via* binding to CD112R (46, 47) and not *via* TIGIT (48). Therefore, it is reasonable to consider that modulation of T cell and NK cell functions *via* TIGIT is mainly by mediated by interaction with CD155.

## The Mechanisms of TIGIT Co-Inhibition

**Figure 1:**
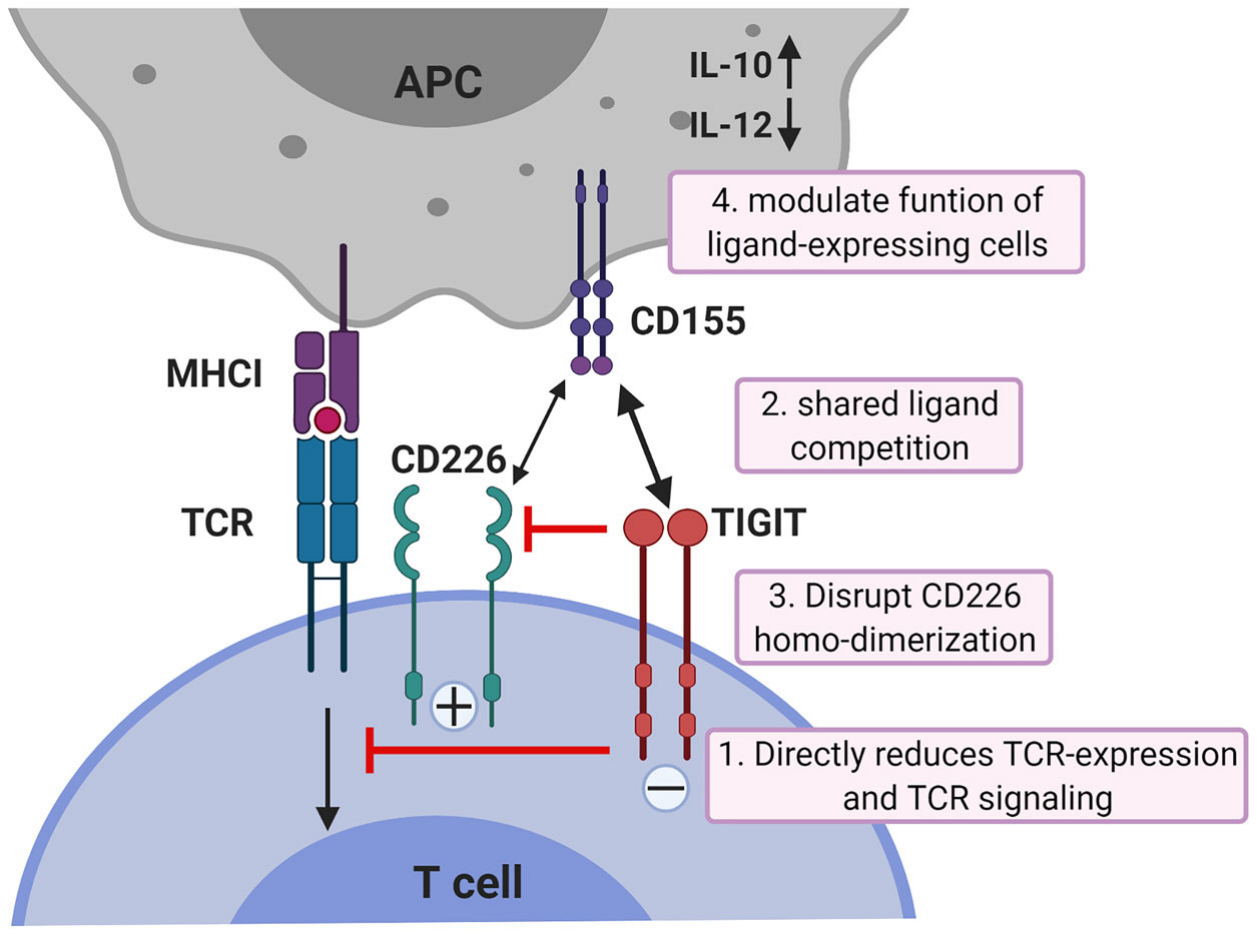
Mechanisms of TIGIT inhibition in T cells. TIGIT displays multiple inhibitory mechanisms in T cells. 1) TIGIT binds to CD155 and delivers intracellular inhibitory signals which directly reduces TCR-expression and TCR signaling. 2) TIGIT binds to CD155 with much higher affinity than its co-stimulatory counterpart CD226 and thereby can replace CD226 from CD155 binding; 3) or disrupts CD226 homo-dimerization to inhibit CD226-mediated T cell activation. 4) TIGIT binds to CD155 on APCs to induce IL-10 production and decrease IL-12 production which indirectly inhibits T cells. APCs, antigen-presenting cells. The figure is adapted based upon the cartoon from Pauken KE et al. (53).

While all co-inhibitory receptors have the ability to suppress T cell activation, they differ in potency, kinetics of expression and with respect to the cellular signaling pathways they alter. Whereas the co-inhibitory checkpoint CTLA-4 acts downstream of TCR-induced signaling by targeting downstream effectors of PI3K through activation of the serine/threonine phosphatase PP2A (49), TIGIT acts more upstream (50). TIGIT can inhibit CD8^+^ T cell proliferation and activation by directly acting on TCR expression itself as engagement of TIGIT induces a down-regulation of the TCR-α chain and molecules that comprise the TCR complex (18). In addition, TIGIT can reduce TCR-induced p-ERK signaling in CD8^+^ T cells (50). Binding of TIGIT on NK cells to its ligand CD155 suppresses NK-cell mediated cytotoxicity and IFN-γ production through signaling cascades generated by ITIM and ITT-like motifs in its cytoplasmic tail (22–24). However, TIGIT exerts its functions not only by direct cell-intrinsic inhibitory signaling, but like CTLA4 which blocks binding of its co-stimulatory counterpart CD28 to their shared ligands CD80 and CD86, also in an indirect way. TIGIT can compete for ligand binding with CD226 thereby reducing T-cell co-stimulation *via* CD226 (51). In addition, TIGIT can prevent co-stimulatory signaling *via* CD226 by blocking CD226 homo-dimerization (52). Finally, TIGIT can suppress T-cells indirectly by modulating functions of cells expressing its ligand CD155. TIGIT expressed on CD4^+^ T cells induces IL-10 and suppresses IL-12 production by DCs *via* CD155 ligation and thereby inhibits CD4^+^ T cell proliferation and IFN-γ production (21). The mechanisms of TIGIT co-inhibition of T cells are illustrated in **Figure 1**.

## TIGIT’s Role in Anti-Cancer Immunity

TIGIT is expressed on human tumor-infiltrating CD8^+^ T cells, NK cells, Th and Treg cells in melanoma (54, 55), NSCLC (56, 57), colon cancer (52), HCC (31, 58), gastric cancer (59), glioblastoma (60) and hematological malignancies (42, 61, 62). Increased numbers of intra-tumoral TIGIT^+^CD4^+^ and CD8^+^ T cells are associated with inferior patient outcomes and poor survival in follicular lymphoma patients (61). High TIGIT expression on peripheral CD8^+^ T cells is associated with primary refractory disease in acute myelogenous leukemia (AML) patients (62). Circulating PD-1^+^TIGIT^+^CD8^+^ T-cell populations are negatively correlated with overall survival rate and progression-free survival rates in patients with hepatitis B virus associated HCC (HBV-HCC) (63). These data suggest a suppressive role of TIGIT in anti-tumor immunity in cancer patients. In the next four paragraphs, we will discuss *via* which tumor-infiltrating immune cell populations TIGIT may inhibit anti-tumor immunity, and how such inhibition could be overcome.

### TIGIT Blockade Synergizes With PD-1 Blockade to Enhance Anti-Tumor Effects of Intra-Tumoral CD8^+^ T Cells

Generating CD8^+^ T cell antitumor responses is the major goal of most cancer immunotherapies because it is considered that CD8^+^ T cells not only provide effective immediate cytotoxicity against tumor cells, but also generate immunological memory to sustain anti-tumor immunity. Knockdown of TIGIT was able to restore *in vitro* IFN-γ and TNF-α production by circulating CD8^+^ T cells from AML patients (62). Nevertheless, single TIGIT blockade achieved no or moderate anti-tumor efficacy in experimental tumor models (37, 52, 58, 64) and in enhancing *in vitro* functionality of human tumor-infiltrating CD8^+^ T cells (31). However, since CD155-TIGIT interaction contributes to cancer resistance to PD-1 blockade (35, 36), inhibition of TIGIT may be a promising strategy to increase the efficacy of PD-1 blockade therapy, especially to combat PD-1 inhibitor resistant tumors (37).

TIGIT is coordinately expressed with PD-1 on human and murine CD8^+^ TILs (52, 54, 58, 65, 66), whereas CD226 expression is negatively associated with PD-1 expression on CD8^+^ TILs (31, 54). In mouse tumors, TIGIT marks a dysfunctional subset of CD8^+^ TILs that co-expresses high levels of PD-1 and TIM3 (66, 67). Similarly, co-expression of TIGIT and PD-1 identifies dysfunctional CD4^+^ and CD8^+^ TIL subsets in human B-cell lymphoma (65). We recently showed that in HCC patients TIGIT expression is up-regulated whereas CD226 expression is down-regulated on CD8^+^ TILs that display high PD-1 expression. These TIGIT^+^PD-1^hi^CD8^+^ T cells co-express exhaustion markers TIM3 and LAG3, show functional defects in IFN-γ and TNF-α production, and demonstrate higher TOX expression and lower TCF-1 expression compared to TIGIT^+^PD-1^int^ CD8^+^ TILs, together characterizing them as exhausted dysfunctional CD8^+^ TILs (31). While CD8^+^PD-1^int^TIGIT^+^ T cells are found in tumors of all HCC patients, TIGIT-expressing CD8^+^PD-1^hi^ TILs are present in a subset of HCC patients (31) and are associated with poor prognosis (68), supporting their poor anti-tumor functions. Interestingly, it has been demonstrated that *in vitro* PD-1 blockade upregulates TIGIT expression on NY-ESO-1-reactive CD8^+^ T cells from melanoma patients (54), and that TIGIT is the most-upregulated immune checkpoint on CD8^+^ TILs upon anti-PD-1 treatment in a PD-1 non-responsive HCC mouse model (37), suggesting TIGIT as a plausible target to improve efficacy of anti-PD-1 treatment.

In the MC38 colon carcinoma mouse model, TIGIT blockade alone led to a small but uniform retardation of tumor growth. Anti-PD-1 treatment alone resulted in a variable response with most mice showing initial tumor regression followed by escape and only one mouse showing complete tumor regression. However, anti-TIGIT/anti-PD-1 co-blockade increased IFN-γ, TNF-α and IL-2 in CD8^+^ TILs and resulted in complete tumor regression in all mice (69). Likewise, in the CT26 colon carcinoma (52) and glioblastoma (64) mouse models, co-blockade of TIGIT and PD-1/PD-L1 pathway synergistically decreased tumor burden and improved survival by increasing IFN-γ^+^ CD8^+^ TILs (52). In HCC-bearing mice which were resistant to PD-1 blockade, dual blockade of TIGIT and PD-1 expanded the effector memory CD8^+^ T cell population and increased ratio of cytotoxic T cells to Treg in tumors, resulting in suppressed tumor growth and prolonged survival (37). Combined PD-1/TIGIT blockade resulted also in another mouse HCC model in synergistic inhibition of tumor growth and significantly prolonged mice survival (58). Furthermore, co-blockade of PD-1 and TIGIT increased the *in vitro* proliferation, cytokine production and anti-tumor function of CD8^+^ TILs from HCC and melanoma patients (31, 54).

The balance of TIGIT/CD226 expression on CD8^+^ T cells is important for proper CD8^+^ function, and the effect of TIGIT blockade is abrogated by CD226-blockade (31, 52, 70, 71), demonstrating that co-stimulation *via* CD226 is required for stimulatory effect of TIGIT blockade on CD8^+^ T cells. CD226^hi^ CD8^+^ T cells but not CD226^lo^ CD8^+^ T cells are required for anti-TIGIT responses (71). Remarkably, CD226 is also required for enhancement of anti-tumor CD8^+^ T-cells responses by PD-1 blockade. Anti-PD-1 cancer treatment efficacy is hampered in CD226^-/-^ mice (38, 72). Moreover, numbers of CD226^+^CD8^+^ TILs in pre-treatment tumor biopsies of melanoma patients are associated with improved progression-free survival following single anti-PD-1 therapy or anti-PD-1/anti-CTLA4 co-blockade (38). Unfortunately, CD226 expression is down-regulated on CD8^+^ TILs in mouse and human tumors (38, 54, 71, 72), especially on CD8^+^PD-1^hi^ exhausted dysfunctional CD8^+^ TILs (31) which have been shown to contain the majority of tumor-reactive cells (57, 73). As mentioned above, down-regulation of CD226 on CD8^+^ T cells in tumors is driven by its engagement with CD155 which causes internalization and proteasomal degradation of CD226 (38) but additionally, PD-1 signaling suppresses CD226 signaling (70). These mechanisms may explain the limited effect of single TIGIT blockade on CD8^+^ TIL anti-tumor functions. Interestingly, blockade of PD-1 restores CD226 co-stimulatory signaling (70), and this observation may explain the synergy between PD-1 and TIGIT blockade in enhancing CD8^+^ TIL responses.

**Figure 2:**
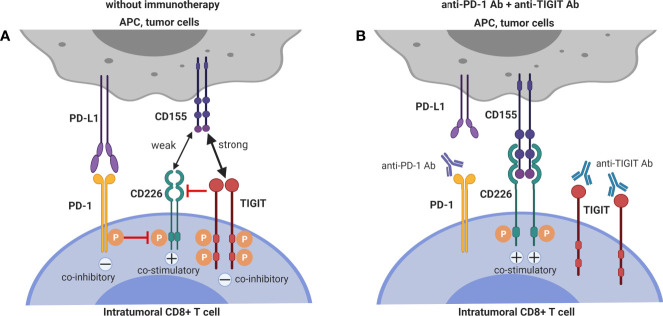
Dual blockade of TIGIT and PD-1 has synergistic effects on intra-tumoral CD8+ T cells. **(A)** CD226 co-stimulatory signaling is suppressed by both TIGIT and PD-1 signaling. **(B)** Co-blockade of PD-1 and TIGIT synergizes to restore CD226 co-stimulatory signaling.

Together with pre-clinical (35, 37, 38) and clinical data (36) showing that CD155 expression on tumor cells contributes critically to resistance to anti-PD-1 immunotherapy, the pre-clinical data that demonstrate and mechanistically explain synergy between TIGIT-blockade and PD-1 blockade to enhance anti-tumor CD8^+^ T cell immunity, argue that dual PD-1 and TIGIT blockade is a promising combinatorial approach to overcome resistance to PD-1/PD-L1 single blockade. The interactions between the mechanisms of T cell co-inhibition by TIGIT and PD-1 interactions are depicted in **Figure 2**.

### Blockade of TIGIT on Tregs Reduces Their Immunosuppressive Functions

**Figure 3:**
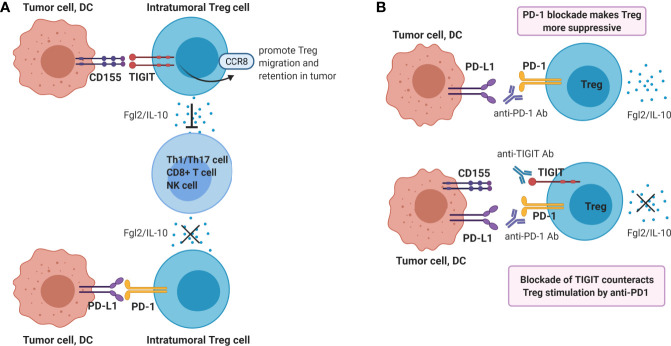
Ligation of TIGIT on Tregs contributes to dampening of anti-tumor immunity. **(A)** Ligation of TIGIT augments suppressive function of Tregs, which inhibits Th1/17 cell, CD8^+^ T cell or NK responses *via* production of Fgl2 and IL-10. On the contrary, PD-1 ligation may reduce Treg suppressive function. **(B)** Single blockade of PD-1 may enhance Treg immunosuppression, whereas dual blockade of TIGIT and PD-1 may counteract this resistance mechanism.

Tregs play a dominant role in the regulation of antitumor immunity, and suppress multiple steps of the cancer immunity cycle. In contrast to the dampening action that TIGIT ligation has on CD8^+^ T- and NK cell functions, the suppressive function of Treg is enhanced by engagement of TIGIT on Treg. TIGIT is constitutively expressed in most Tregs in humans (31, 74–76) and mice (74), and agonistic TIGIT ligation augments the suppressive function and stability of these cells (74, 76). Conversely, agonistic ligation of CD226 on Tregs disrupts their suppression (76). High TIGIT expression marks highly active suppressive Tregs. Compared to TIGIT^-^ Tregs, TIGIT^+^ Tregs express higher levels of FoxP3, co-inhibitory molecules (ICOS, CTLA4, PD-1 and TIM3) as well as the immunosuppressive cytokine IL-10, indicating that TIGIT^+^ Treg might be optimally equipped for mediating immunosuppression (67, 74, 76). TIGIT^+^ Treg specifically suppress pro-inflammatory T helper 1 (Th1) and Th17 cells, but not Th2 cell responses, and ligation of TIGIT on Treg induces production of fibrinogen-like protein 2 which suppresses effector T cell proliferation (74). Additionally, TIGIT signaling in Tregs upregulates CCR8 which may promote Treg migration and retention in tumor tissue (67, 77, 78) (**Figure 3A**).

TIGIT^+^ Treg have been demonstrated to accumulate both in mouse tumors (67) and in several types of human tumors (76, 79). In HCC TILs, Treg represent the population of lymphocytes with highest TIGIT expression (31). In contrast, CD226 is down-regulated on tumor-infiltrating Treg, and a high TIGIT/CD226 expression ratio on tumor-infiltrating Tregs correlates with high Treg frequencies in tumors and poor clinical outcome in melanoma patients treated with anti-PD-1 and/or anti-CTLA4 immune checkpoint blockade, suggesting that the TIGIT/CD226 ratio in Tregs is a marker of Treg stability (76). In an *in vitro* study, TIGIT blockade depleted FoxP3^+^ Tregs while increasing proliferation of IFNγ-producing CD4^+^ T cells from peripheral blood from patients with multiple myeloma (42). Adoptive transfer experiments in mouse tumor models have even suggested that TIGIT ligation dampens anti-tumor immunity predominantly *via* regulatory T cells, rather than through a direct effect on TIGIT^+^CD8^+^ T cells (67). However, a study in which different T-cell subsets were depleted by specific antibodies found that CD8^+^ T cell depletion as well as Treg depletion could abrogate the anti-tumor effects of TIGIT-blockade in a mouse cancer model (66).

Whether PD-1-blockade might synergize with TIGIT-blockade in reducing suppression of intra-tumoral immunity Treg has not yet been investigated. The functional effects of PD-1 expression on Tregs is still a matter of debate. On the one hand, PD-1-deficient mouse Tregs have an enhanced suppressive capacity (80) and *in vitro* PD-L1 blockade augmented the suppressive capacity of Treg from HCV-infected patients (81). Moreover, PD-1 blockade enhanced the suppressive function PD-1^+^ Treg isolated from human tumors (82). On the other hand, interaction between PD-1 on Tregs from mice with chronic LMCV-infection with PD-L1 expressed on CD8^+^ T cells was found to contribute to their capacity to suppress CD8^+^ T cell functions (83) and blockade of PD-L1 has been shown to reduce the *in vitro* suppressive function of human tumor-infiltrating Treg (79).

Therefore, PD-1/PD-L1 blockade may either enhance or reduce the suppressive capacity of PD-1-expressing Treg in the tumor micro-environment. In both cases, co-blockade of TIGIT may be beneficial. If PD-1/PD-L1 blockade enhances intra-tumoral Treg immunosuppression, this mechanism may serve as a resistance mechanism against PD-1/PD-L1 blockade, which is supported by recent data in cancer patients treated with anti-PD-1 antibody (82). In this case, co-treatment with a blocking anti-TIGIT antibody may counteract this resistance mechanism (**Figure 3B**). Alternatively, if PD-1 and TIGIT expressed on chronically activated Treg present within the tumor micro-environment both contribute to their strong suppressive capacity, PD-1 blockade may synergize with TIGIT-blockade in reducing local immune-suppression exerted by tumor-infiltrating Treg functions. Both mechanisms may contribute to the synergistic effect of dual TIGIT/PD-1 blockade on anti-tumor immunity observed in pre-clinical models, and both hypotheses are worth to be investigated.

### The Role of TIGIT in Regulation Of Memory CD4^+^ T Cells in Cancer Is Under-Investigated

The requirement for CD4^+^ T cells in anti-tumor responses has been attributed to providing help during priming to achieve full activation and effector function of tumor-specific CD8^+^ T cells (84). Mouse TIGIT^–/–^ CD4^+^ T cells showed increased proliferation upon re-stimulation with antigenic peptide, and produced higher amounts of IFN-γ and lower amounts of IL-10, indicating a T cell intrinsic opposite effect in regulating IFN-γ versus IL-10 production in CD4^+^ T cells (18). Moreover, the interaction of TIGIT on mouse Th with CD155 on DCs induced phosphorylation of CD155 and consequently upregulation of IL-10 production and downregulation of IL-12 production by DC, which suggests the existence of an IL-10-driven feedback loop for TIGIT-mediated Th cell suppression (21). TIGIT is expressed on around 20% of intra-tumoral Th in HCC patients, but not over-expressed compared to Th in tumor-free liver tissue and in the circulation (31). Similar to CD8^+^ T cells and Treg, CD226 is downregulated on intra-tumoral Th in HCC patients. In contrast, TIGIT is highly expressed on effector memory (around 80%), effector (around 50%) and central memory (around 40%) CD4^+^ T cells in follicular lymphoma patients (50). PD-1^+^TIGIT^+^ CD4^+^ T cells from non-Hodgkin lymphoma patients produced lower amounts of IL2, IFN-γ and TNF-α compared to other subsets after re-stimulation (65), suggesting loss of poly-functionality. Indeed, tumor-infiltrating CD4^+^ T cells expressing IL2 were increased after TIGIT blocking monoclonal antibody (mAb) treatment in a head and neck squamous cell carcinoma mouse model (66). Moreover, animals treated with dual anti-TIGIT/PD-1 antibodies showed increased IFN-γ secretion by CD4^+^ TILs in the MC38 colon tumor mouse model, compared to single anti-PD-1 treatment (69). There are few studies which investigated TIGIT function in regulation of T helper cells in tumors, and this requires further investigation.

### Blockade of TIGIT on NK Cells Augments Anti-Tumor Immunity

NK cells not only detect and identify malignant cancer cells, but they also induce cancer cell death (85, 86), e.g. by destroying MHC class I-deficient tumor cells which are refractory to CD8^+^ T cell-mediated immunity. In contrast, lack of MHC class I expression renders cells vulnerable to NK cell killing, because they are devoid of a major NK-cell inhibitory mechanism exerted by binding of self-MHC class I to inhibitory Killer Immunoglobulin Receptors (KIR) and NKG2A/CD94. Interestingly, defects in MHC class I presentation are relatively frequent in cancer cells. Moreover, mutations in β2-microglobulin which cause lack of surface MHC class I expression are also involved in acquired resistance to anti-PD-1 therapy (87, 88). For these tumors, appropriate activation of NK cells in the tumor microenvironment may be a promising alternative therapeutic option (89). In addition, NK cells help to trigger a more robust anti-tumor T-cell response by engaging other immune cells (90, 91). For example, CD8^+^ T cell responses against tumor cells are stimulated by NK-initiated cytokine production (91–93).

TIGIT expression on tumor-infiltrating NK cells is associated with tumor progression and is linked to functional exhaustion of NK cells in multiple cancer models (94). NK cell functions are regulated by the net balance of signals perceived by their activating and inhibiting receptors (95). The CD226 activating receptor and the TIGIT and CD96 inhibitory receptors have been shown to be key regulators of anti-tumor immune responses by NK cells. They share the same ligand CD155 (96). In this review, we will focus on the role of TIGIT in regulating anti-tumor functions of NK cells. In the B16 melanoma mouse model, NK cell-specific TIGIT-deficiency led to improved survival. Additionally, it induced a lower frequency of tumor-infiltrating CD8^+^ T cells expressing TIGIT and TIM3, which suggested that NK cells expressing TIGIT might also indirectly contribute to the exhaustion of CD8^+^ T cells (94). Blockade of TIGIT inhibited tumor growth and prevented exhaustion of tumor-infiltrating NK cells in the CT26 colon carcinoma mouse model (94). Mechanistically, tumor cells expressing CD155 impair NK cell functions by downregulating their CD226 expression (97, 98). CD155-/- mice displayed reduced tumor growth and metastasis *via* CD226 upregulation and enhanced effector functions of NK cells (35). Similarly, NK cell-specific TIGIT-deficiency resulted in a greater frequency of intra-tumoral NK cells expressing CD226 in the B16 melanoma mouse model (94). Furthermore, antibody blockade of nectin-4 suppresses human tumor growth in SCID mice transplanted with human NK cells, while nectin-4 overexpression in human tumor cells led to increased tumor growth in the presence of human NK cells (25). However, whether TIGIT blockade synergizes with PD-1 blockade to improve NK cell cytotoxicity is as yet unknown. Notably, IL-15 can increase the expression of both CD226 and TIGIT by intra-tumoral NK cells (98), and combination therapy of IL-15 and TIGIT blockade increased cytotoxicity of NK cells against melanoma and suppressed lung metastasis in mouse melanoma models. The authors suggest the development of novel combinatorial immunotherapy with IL-15 and TIGIT blockade to promote NK cell-mediated destruction of MHC class I-deficient melanoma, which are refractory to CD8^+^ T cell-mediated immunity (98).

**Figure 4:**
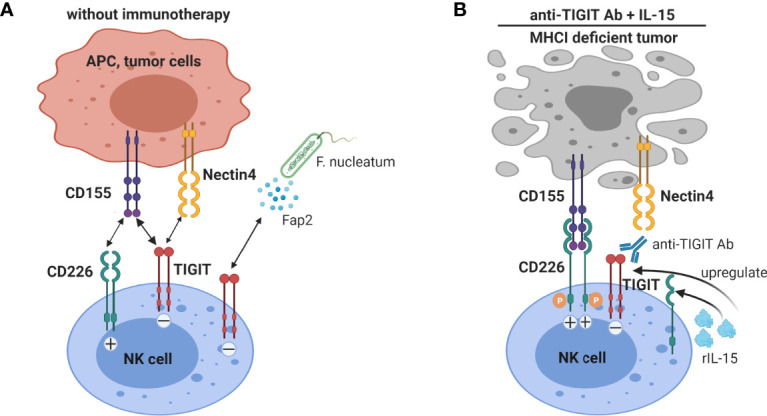
Blockade of TIGIT on NK cells augments anti-tumor immunity. **(A)** NK cells expressing TIGIT are functionally impaired by binding to CD155 and nectin-4, and in colorectal cancer to Fap2 protein produced by a gut bacterium. **(B)** TIGIT blockade not only interrupts inhibitory signaling by TIGIT in NK cells, but also allows interaction of the co-stimulatory receptor CD226 with CD155. IL-15 treatment increases TIGIT and CD226 expression on NK cells. Thus IL-15 combined with TIGIT blockade further enhanced NK cells anti-tumor functions, especially promoting NK cell-mediated destruction of MHC class I-deficient tumors.

In humans TIGIT was shown to have variable expression on NK cells, and high TIGIT expression correlated with reduced NK cell functions (99). A TIGIT/CD226 dysbalance with high TIGIT and low CD226 expression on intra-tumoral NK cells been shown in multiple human tumors, e.g. colorectal cancer (CRC) (98), ovarian cancer (100) and HCC (101), and compromises intra-tumoral NK cell functions. *In vitro* blockade of the TIGIT pathway significantly increased functions of circulating NK-cells, particularly in NK cells with high levels of TIGIT expression (99). TIGIT^+^ NK cells from metastatic melanoma patients displayed a lower cytolytic activity than TIGIT^–^ NK cells against CD155^+^ MHC class I-deficient melanoma cells (98). TIGIT blockade boosts *in vitro* functional responsiveness of ovarian cancer ascites-derived CD56^dim^ NK cells in patients with baseline reactivity against ovarian cancer tumor cells (100). Blocking TIGIT was shown to increase circulating NK cell cytotoxicity against HCC cell lines, suggesting that targeting TIGIT may be a beneficial approach to improve NK cell function in HCC patients (101). Gur C et al. showed that a CRC-associated bacterium (Fusobacterium nucleatum) inhibits human NK cells *via* TIGIT, which can be blocked by anti-TIGIT antibody (102), suggesting that NK-cell responses in CRC can be suppressed by the gut microbiome in a TIGIT-mediated fashion. Collectively, TIGIT blockade has convincingly been shown to enhance anti-tumor NK cell responses. The interaction of TIGIT on NK cells with its ligands and the effect of anti-TIGIT and IL-15 co-treatment on NK cells are illustrated in **Figure 4**.

## TIGIT in Clinical Trials to Treat Cancer

Over last two years, the above mentioned immunomodulatory effects of TIGIT have attracted the attention from pharmaceutical companies worldwide. Currently, nine human anti-TIGIT mAbs are being tested in 43 phase 1/2/3 clinical trials either as a monotherapy or, in most studies, in combination with anti-PD-1/PD-L1 antibodies or chemotherapies for the treatment of advanced solid tumors, and in two trials in multiple myeloma (**Table 1**). Recently, in a phase I study anti-TIGIT antibody vibostolimab was demonstrated to be well tolerated as monotherapy and in combination with pembrolizumab (anti-PD-1 antibody), achieving antitumor activity in patients with advanced NSCLC naïve or refractory to anti-PD-1/PD-L1 therapy (NCT02964013) (103, 104). The first data of the clinical efficacy of TIGIT mAbs have been reported as well. Interim data from an ongoing global phase II trial (CITYSCAPE, NCT03563716) demonstrated the improved efficacy of an anti-TIGIT antibody tiragolumab in combination with anti-PD-L1 antibody atezolizumab compared to atezolizumab alone in PD-L1-positive metastatic NSCLC patients in terms of overall response and progression free survival (105). Importantly, all grade 3-5 adverse events (AEs) were similar in both groups (48% *vs* 44%) (105), in accordance with pre-clinical data showing limited immunotoxicity in TIGIT-deficiency in mice, as discussed earlier.

**Table 1:** Currently ongoing clinical trials involving human anti-TIGIT mAbs.

Agent	Trial sponsor	ClinicalTrials.gov identifier	Type of trial	Estimated enrollment	Tumor type	Combined therapy
BMS-986207	Bristol-Myers Squibb	NCT02913313	Phase 1/2	170	Advanced solid tumors	Monotherapy or combined with nivolumab (anti-PD-1 mAb)
	Multiple Myeloma Research Consortium	NCT04150965	Phase 1/2	104	Refractory multiple myeloma	Monotherapy or combined with pomalidomide and dexamethasone
	Compugen Ltd	NCT04570839	Phase 1/2	100	Advanced solid tumors	Combined with Nivo and COM701(inhibitor of poliovirus receptor)
	Genentech/Roche	NCT03563716	Phase 2	135	Locally advanced or metastatic Non-small cell lung cancer (NSCLC)	Combined with atezolizumab (anti-PD-L1 mAb)
Tiragolumab (MTIG7192A; RG6058)	Roche	NCT04294810	Phase 3	500	Locally advanced or metastatic PD-L1-selected NSCLC	Combined with atezolizumab
		NCT04513925	Phase 3	800	NSCLC stage III	Combined with atezolizumab compared with Durvalumab
		NCT04619797	Phase 2	200	Unresectable or metastatic NSCLC	Combined with atezolizumab and pemetrexed
		NCT04256421	Phase 3	400	Untreated extensive-stage small cell lung cancer (SCLC)	Combined with atezolizumab and carboplatin and etoposide (CE)
		NCT04308785	Phase 2	363	SCLC	Combined with atezolizumab VS atezolizumab
		NCT03281369	Phase 1b/2	410	Esophageal cancer	Combined with atezolizumab VS atezolizumab and chemotheraoy
		NCT04540211	Phase 3	450	Unresectable esophageal cancer	Combined with atezolizumab +paclitaxel and cisplatin (PC) VS placebo+PC
		NCT04543617	Phase 3	750	Unresectable esophageal squamous cell carcinoma	Combined with atezolizumab VS atezolizumab
		NCT04524871	Phase 1/2	100	Advanced liver cancers	Combined with Atezolizumab + Bevacizumab
		NCT04584112	Phase 1	80	Triple-negative breast cancer	Combined with atezolizumab + chemotherapy
		NCT04045028	Phase 1	52	Refractory multiple myeloma	Monotherapy or combined with daratumumab or rituximab
		NCT02794571	Phase 1	540	Locally advanced or metastatic tumors	Monotherapy or in combined with atezolizumab and/or other anti-cancer therapies
		NCT04300647	Phase 2	220	Cervical cancer (PD-L1-positive)	Combined with atezolizumab
		NCT03281369	Phase 1/2	410	Gastric and esophageal cancer	Combined with atezolizumab with/without cisplatin+5FU
		NCT03193190	Phase 1/2	290	Metastatic pancreatic ductal Adenocarcinoma	Combined with atezolizumab +chemotherapy
		NCT03869190	Phase 1/2	385	Locally advanced or metastatic urothelial carcinoma	Combined with atezolizumab
Tiragolumab (MTIG7192A; RG6058)	Roche	NCT04665843	Phase 2	120	PD-L1-positive squamous cell carcinoma of the head and neck	Combined with atezolizumab
		NCT03708224	Phase 2	55	Squamous cell carcinoma of the head and neck	Combined with atezolizumab VS atezolizumab
MK-7684	Merck	NCT02964013	Phase 1	492	Advanced solid tumors	Monotherapy or combined with pembrolizumab (anti-PD-1 mAb)
		NCT04305041	Phase 1/2	200	Melanoma	Combined with pembrolizumab VS pembrolizumab
		NCT04305054	Phase 1/2	135	Melanoma	Combined with pembrolizumab VS pembrolizumab
		NCT04738487	Phase 3	598	PD-L1 positive metastatic NSCLC	Combined with pembrolizumab VS pembrolizumab
		NCT04725188	Phase 2	240	Metastatic NSCLC	Combined with pembrolizumab +Docetaxel VS Docetaxel
		NCT04165070	Phase 2	90	Advanced NSCLC	Combined with pembrolizumab + Carboplatin + Paclitaxel
		NCT02861573	Phase 1	1000	Metastatic castrate resistant prostate cancer	Combined with pembrolizumab
		NCT04303169	Phase 1/2	65	Stage III melanoma	Combined with pembrolizumab VS pembrolizumab
AB154	Arcus Biosciences	NCT03628677	Phase 1	66	Advanced solid tumors	Monotherapy or combined with AB122 (anti-PD-1 mAb)
		NCT04262856	Phase 2	150	NSCLC	Combined with AB122 or AB122 and AB928 (dual adenosine receptor antagonist)
		NCT04656535	Phase 1	46	Recurrent glioblastoma	Combined with AB122
		NCT04736173	Phase 3	625	PD-L1 positive metastatic NSCLC	Combined with AB122 VS AB122
COM902	Compugen	NCT04354246	Phase 1	45	Advanced malignant tumors	Monotherapy
IBI939	Innovent Biologics	NCT04353830	Phase 1	270	Advanced malignant tumors	Monotherapy or combined with sintilimab (anti-PD-1 mAb)
		NCT04672356	Phase 1	20	Advanced lung cancer	Combined with sintilimab
		NCT04672369	Phase 1	42	Advanced NSCLC	Combined with sintilimab VS sintilimab
BGB-A1217	BeiGene	NCT04047862	Phase 1	39	Advanced solid tumors	Monotherapy or combined with tislelizumab (anti-PD-1 mAb)
		NCT04732494	Phase 2	280	Recurrent or metastatic Esophageal squamous cell carcinoma	Combined with tislelizumab VS tislelizumab +placebo
		NCT04746924	Phase 3	605	Locally advanced or metastatic NSCLC	Combined with tislelizumab VS pembrolizumab+placebo
		NCT04693234	Phase 2	167	Metastatic cervical cancer	Combined with tislelizumab VS tislelizumab
ASP8374	Astellas Pharma	NCT03260322	Phase 1	169	Advanced solid tumors	Monotherapy or combined with pembrolizumab (anti-PD-1 mAb)
		NCT03945253	Phase 1	6	Advanced solid tumors	Monotherapy
M6223	EMD Serono	NCT04457778	Phase 1	35	Advanced solid tumors	Monotherapy or combined with Bintrafusp alfa

## Concluding Remarks

Therapeutic immune checkpoint inhibition has revolutionized cancer treatment, and recent experience has made clear that combination therapies that target multiple CPI are more effective than monotherapies. TIGIT has been shown to be expressed by dysfunctional CD8^+^ T cells and NK cells, as well as highly immunosuppressive regulatory T cells in mouse and human tumors. Its main ligand CD155 is over-expressed in several types of cancer and hampers immune surveillance by interacting with TIGIT on these immune cells. Preclinical studies have demonstrated that blocking TIGIT-signaling improves CD8^+^ T-cell and NK-cell responses and reduces suppressive functions of regulatory T cells. However, single TIGIT-blockade has minimal effects on tumor growth in most experimental tumor models, and is also insufficient to reinvigorate functions of human tumor-infiltrating CD8^+^ T cells. TIGIT-blockade synergizes with PD-1/PDL-1 blockade to enhance anti-tumor CD8^+^ T-cell immunity in pre-clinical models, and is even effective in mouse tumors models that are resistant of PD-1 blockade, thus providing hope for clinical efficacy in patients with anti-PD-1 resistant tumors. A unique property of TIGIT among inhibitory immune checkpoints is that its blockade not only augments anti-tumor effector CD8^+^ T-cell responses, but also anti-tumor NK-cell responses, and reduces the suppressive capacity of regulatory T cells. Whether TIGIT blockade can enhance T-helper cell responses it largely unknown. The clinical proof of its efficacy, especially for cancers that are resistant against single anti-PD-1 therapy, in ongoing clinical trials is eagerly awaited for.

## Author Contributions

ZG wrote the initial draft of the manuscript and made figures. MP contributed to outline and revision of the manuscript. DS and JK contributed to writing and revision of the manuscript. All authors contributed to the article and approved the submitted version.

## Funding

This study was supported by the China Scholarship Council which provided a PhD fellowship grant to ZG (number 201606230253).

## Conflict of Interest

The authors declare that the research was conducted in the absence of any commercial or financial relationships that could be construed as a potential conflict of interest.

## Abbreviations

TIGIT, T cell immunoglobulin and ITIM domain; ITIM, immunoreceptor tyrosine-based inhibitory motif; PD-1, Programmed cell Death protein 1; TIM3, T-cell immunoglobulin and mucin-domain containing-3; LAG3, Lymphocyte-activation gene 3; CTLs, Cytotoxic T lymphocytes; ICIs, immune checkpoint inhibitors; mAb, monoclonal antibody.
